# Pattern of Adiponectin, Osteocalcin, Irisin, FGF-21, and MCP-1 According to the Body Size Phenotype: Could They Be Markers of Metabolic Health in Mexican-Mestizo Middle-Aged Women?

**DOI:** 10.3390/metabo11110771

**Published:** 2021-11-11

**Authors:** Lourdes Balcázar-Hernandez, Lourdes Basurto, Leticia Manuel-Apolinar, Sara Vega-García, Norma Basurto-Acevedo, Carlos Martínez-Murillo, Rosalinda Sánchez-Arenas

**Affiliations:** 1 Endocrinology Department, Hospital de Especialidades, Centro Médico Nacional Siglo XXI, Instituto Mexicano del Seguro Social, Av. Cuauhtémoc 330, Doctores, Mexico City CP 06720, Mexico; dra.lourdesbalcazar@gmail.com; 2 Endocrine Research Unit, Centro Medico Nacional Siglo XXI, Instituto Mexicano del Seguro Social, Av. Cuauhtémoc 330, Doctores, Mexico City CP 06720, Mexico; letymanu@yahoo.com.mx (L.M.-A.); sarave3@yahoo.com (S.V.-G.); 3 Surgical Department, Hospital Angeles Metropolitano, Tlacotalpan 59, Roma Sur, Cuauhtémoc, Mexico City CP 06760, Mexico; normabasurtoa@outlook.com; 4 Hematology Department, Hospital General de México, Dr. Balmis 148, Doctores, Cuauhtémoc, Mexico City CP 06720, Mexico; carlosmtzmurillo@gmail.com; 5 Epidemiology and Health Services Research, Centro Médico Nacional Siglo XXI, Instituto Mexicano del Seguro Social, Av. Cuauhtémoc 330, Doctores, Mexico City CP 06720, Mexico; felicitasarenas@gmail.com

**Keywords:** adiponectin, osteocalcin, irisin, FGF-21, MCP-1, obesity, metabolic phenotype

## Abstract

Variations in levels of some adipokines, myokines, osteokines, hepatokines and inflammatory cytokines contribute to abnormal glucose and lipid metabolism. The aim of this study was to determine the pattern of adiponectin, osteocalcin (OCN), irisin, FGF-21, and MCP-1 according to the body size phenotype of middle-aged women, and their associations with BMI, visceral adipose tissue (VAT), and HOMA-IR. A cross-sectional study in 265 women aged from 40 to 65 years was performed. The biochemical characteristics were evaluated in metabolically healthy normal weight, metabolically unhealthy normal weight, metabolically healthy obese, and metabolically unhealthy obese women. There was an association of OCN with BMI (r = −0.107; *p* = 0.047); adiponectin with BMI (r = −0.217; *p* = 0.001), insulin (r = −0.415; *p* = 0.0001), HOMA-IR (r = −0.429; *p* = 0.0001), and VAT (r = −0.134; *p* = 0.025); irisin with BMI (r = 0.604; *p* = 0.001), insulin (r = 0.446; *p* = 0.0001), HOMA-IR (r = 0.452; *p* = 0.0001), and VAT (r = 0.645; *p* = 0.0001); FGF−21 with insulin (r = −0.337; *p*= 0.030) and HOMA-IR (r = −0.341; *p* = 0.03); and MCP-1 with BMI (r = 0.481; *p* = 0.0001), VAT (r = 0.497; *p* = 0.001), insulin (r = 0.298; *p*= 0.001), and HOMA-IR (r = 0.255; *p* = 0.004). A multivariate analysis showed that an elevation of OCN (OR 1.4 (_95%_CI 1.06–1.81)) and a reduction of adiponectin (OR 0.9 (0.84–0.96)) were associated factors for a metabolic unhealthy phenotype in normal weight participants. Likewise, higher irisin (OR 1.007 (1.003–1.011)) and MCP-1 (1.044 (1.008–1.083)) were risk factors for a metabolic unhealthy phenotype in woman with obesity. OCN, adiponectin, irisin, FGF-21, and MCP-1 are associated with some metabolic parameters such as BMI, HOMA-IR, and VAT, and could be possible biomarkers of an unhealthy metabolic phenotype in middle-aged women.

## 1. Introduction

Some cytokines play an important role in metabolic homeostasis. The mechanisms involved are not entirely clear, but their alterations are associated with lipid accumulation, changes in energy metabolism and an inflammatory profile. These cytokines include: adipokines (e.g., leptin, adiponectin, resistin, omentin-1, asprosin), myokines (e.g., irisin, IL-13, IL-15), osteokines (e.g., osteocalcin (OCN), osteopontin, esclerostin, fibroblast growth factor-23 (FGF-23)), hepatokines (e.g., fibroblast growth factor-21(FGF-21), hepassocin, fetuin A and B, selenoprotein P), and inflammatory cytokines (e.g., tumor necrosis alpha (TNF-α), interleukin-1β (IL-1β), interleukin-6 (IL-6), and monocyte chemoattractant protein-1 (MCP-1)) [1].

Previously, Raschke and Eckel determined that adipokines and myokines are both mediators of exercise and inflammation. They proposed the term “adipo-myokines” to identify the contraction-regulated myokines that are also secreted by adipocytes. This has led to the study of the role of these cytokines in metabolic disease, in order to develop new prevention and treatment strategies [2]. In the last two decades, the importance of the musculoskeletal system in energy homeostasis has been demonstrated. Several myokines and osteokines interact with adipose tissue to modulate the secretion of adipokines, and these adipokines can also modulate muscle and bone metabolism [3].

Myokines, osteokines, and adipokines exert autocrine, paracrine, and endocrine effects to regulate muscle, bone, fat, and glucose metabolism [3]. It is a well-orchestrated system, so that alterations in one of its components, such as a dysregulation of the energy balance or the visceral accumulation of adipose tissue, lead to a chaotic scenario. Some osteokines also regulate carbohydrate metabolism by modulating pancreatic β-cell function and insulin sensitivity [4].

It is currently known that the alterations in osteokines, myokines, and adipokines, as well as some hepatokines and inflammatory cytokines, contribute to the development of abnormal glucose and lipid metabolism. The elevation of leptin, the reduction of adiponectin, the increased concentrations of FGF-21 and MCP-1, and the lower concentrations of irisin and osteocalcin promote a decreased glucose tolerance and insulin signaling, as well as insulin resistance, β-cell dysfunction, an increased lipolysis, triglyceride synthesis and de novo lipogenesis [1].

Different alterations in adipokines, myokines, osteokines, hepatokines, and inflammatory cytokines have been described in patients with obesity and this may be related to the development of other metabolic diseases [5,6], especially regarding the adipokine adiponectin, the osteokine OCN, the myokine irisin, the hepatokine FGF-21, and the inflammatory cytokine MCP-1 [1]. In the Mexican–Mestizo population, the association of MCP-1 and FGF-21 with the severity of subclinical atherosclerotic disease has been demonstrated in women without cardiovascular disease (CVD) [7]. Likewise, the negative correlation of OCN with BMI, waist circumference, glucose, insulin, and HOMA-IR in older men has been reported [8].

Typically, obesity has been associated with hypertension, dyslipidemia or alterations in carbohydrate metabolism (Metabolically unhealthy obesity (MUO)); however, it has been observed that 10 to 30% of people with obesity do not have these alterations, which has given rise to the term “metabolically healthy obesity” (MHO). Currently, MHO has been defined as the absence of any metabolic disorder or cardiovascular disease (CVD), including type 2 diabetes, dyslipidemia, hypertension, and atherosclerotic cardiovascular disease in a person with obesity [9].

On the other hand, there is a group of individuals with a metabolically unhealthy but normal weight phenotype (body mass index (BMI) 18.5–24.9 kg/m^2^), referred to as a metabolically unhealthy normal weight (MUNW). These individuals are at high risk of CVD [10]. In this group, there is a lack of information about the pattern of adipokines, myokines, osteokines, and hepatokines, as well as their differences when compared with those of a metabolically healthy normal weight (MHNW), MUO, and MHO.

The aim of this study was to determine the pattern of adiponectin, OCN, irisin, FGF-21, and MCP-1 according to the body size phenotype in middle-aged women, to identify the differences between groups and to evaluate the association of these cytokines with BMI, visceral adipose tissue (VAT), insulin and the homeostasis model assessment of insulin resistance (HOMA-IR).

## 2. Results

### 2.1. Clinical and Biochemical Characteristics of Participants

A total of 265 middle-aged women were included. The age of participants were 52.2 ± 5.8 years. According to their metabolic health and the presence or absence of obesity, 6% (*n* = 16) had a MHNW phenotype, 57% (*n* = 151) had a MUNW phenotype, 1.9% (*n* = 5) had a MHO phenotype, and 35.1% (*n* = 93) had a MUO phenotype. Some 67.9% were postmenopausal women. The baseline characteristics of the population are summarized in Table 1.

The values of VAT, cytokines, glucose, insulin, HOMA-IR, and lipid profile are described according to the body size phenotype of the participants in Table 2.

### 2.2. Differences in Adiponectin, OCN, Irisin, FGF-21 and MCP-1 According to the Body Size Phenotype in Middle-Aged Women

#### 2.2.1. MHNW vs. MUNW Phenotype

In normal weighted women, the comparison of the heathy and unhealthy phenotypes showed differences in OCN (3.4 (2.5–5.5) vs. 5.8 (3.7–8.4) ng/mL; *p* = 0.011), adiponectin (16.1 (11.2–21.7) vs. 12.5 (7.9–16.0) µg/mL; *p* = 0.002), glucose (74 (64–85) vs. 83 (77–91) mg/dL; *p* = 0.003), insulin (12.1 (10–13.2) vs. 15.8 (12.6–20.5) mIU/L; *p* = 0.0001), HOMA-IR (2.14 (1.5–2.6) vs. 3.2 (2.4–4.7); *p* = 0.0001], TC [186 (175–190) vs. 230 (211–275) mg/dL; *p* = 0.0001), LDL-C (95 (87–107) vs. 145 (124–178) mg/dL; *p* = 0.0001), HDL-C (67 (54–72) vs. 55 (45–63) mg/dL; *p* = 0.002), and triglycerides (109 (80–125) vs. 145 (109–198) mg/dL; *p* = 0.003) (Table 2). The MUNW phenotype had a higher OCN and lower adiponectin concentrations when compared to the MHNW phenotype.

#### 2.2.2. MHNW vs. MHO Phenotype

In the healthy phenotypes, there were differences found in the OCN (3.4 (2.5–5.5) vs. 5.2 (3.8–6.8) ng/mL; *p* = 0.011), adiponectin (16.1 (11.2–21.7) vs. 14.6 (9.8–29.1) µg/mL; *p* = 0.002), FGF-21 (370 (315–851) vs. 195 (194–197) pg/mL; *p* = 0.024), glucose (74 (64–85) vs. 77 (74–91)mg/dL; *p* = 0.004), insulin (12.1 (10–13.2) vs. 22.5 (12.4–34.4) mIU/L; *p* = 0.0001), HOMA-IR (2.14 (1.5–2.6) vs. 4.2 (2.2–7.9); *p* = 0.0001), TC (186 (175–190) vs. 165 (124.5–178.5) mg/dL; *p* = 0.0001), LDL-C (95 (87–107) vs. 62.6 (54.2–92.6) mg/dL; *p* = 0.0001), HDL-C (67 (54–72) vs. 63.0 (47.5–71.0) mg/dL; *p* = 0.002), and triglycerides (109 (80–125) vs. 121 (88.5–138) mg/dL; *p* = 0.003) between normal weight and obese middle-aged women (Table 2). The MHO phenotype had a higher OCN, with lower adiponectin and FGF-21 concentrations when compared to the MHNW phenotype.

#### 2.2.3. MHNW vs. MUO Phenotype

In the comparison of the MHNW and MUO phenotypes, there were differences found in the VAT (91.5 (68–123) vs. 201 (153–227) cm^2^; *p* = 0.001), adiponectin (16.1 (11.2–21.7) vs. 10.1 (7.6–13.3) µg/mL; *p* = 0.0001), irisin (464 (186–559) vs. 679 (503–787) ng/mL; *p* = 0.0001), FGF-21 (370 (315–851) vs. 162 (21.8–229) pg/mL; *p* = 0.004), glucose (74 (64–85) vs. 89 (79.5–98.5) mg/dL; *p* = 0.001), insulin (12.1 (10–13.2) vs. 22.5 (17.9–30.2) mIU/L; *p* = 0.0001), HOMA-IR (2.14 (1.5–2.6) vs. 4.9 (3.7–7.4); *p* = 0.0001], TC [186 (175–190) vs. 229 (203–262) mg/dL; *p* = 0.0001), LDL-C (95 (87–107) vs. 147 (124–179) mg/dL; *p* = 0.0001), HDL-C (67 (54–72) vs. 49 (41–59) mg/dL; *p* = 0.0001), and triglycerides (109 (80–125) vs. 154 (121–209) mg/dL; *p* = 0.0001) (Table 2). The MUO phenotype had lower adiponectin and FGF-21 concentrations and higher irisin when compared to the MHNW phenotype.

#### 2.2.4. MUNW vs. MUO Phenotype

In participants with an unhealthy phenotype, there were differences in the VAT (105 (87–125) vs. 201 (153–227) cm^2^; *p* = 0.0001), OCN (5.8 (3.7–8.4) vs. 4.7 (3.4–7.0) ng/mL; *p* = 0.0001), adiponectin (12.5 (7.9–16) vs. 10.1 (7.6–13.3) µg/mL; *p* = 0.03), irisin (348.8 (201.4–660) vs. 676 (503–787) ng/mL; *p* = 0.0001), MCP-1 (15.8 (7.7–3.2) vs. 38.6 (32.7–52.8) pg/mL; *p* = 0.0001), glucose (83 (77–91) vs. 89 (79.5–98.5) mg/dL; *p* = 0.007), insulin (15.8 (12.6–20.5) vs. 22.5 (17.9–30.2) mIU/L; *p* = 0.0001), HOMA-IR (3.2 (2.4–4.7) vs. 4.9 (3.7–7.4); *p* = 0.0001), and HDL-C (55 (45–63) vs. 49 (41–59) mg/dL; *p* = 0.01) (Table 2). The MUO phenotype had a lower OCN and adiponectin, as well as a higher MCP-1 and irisin concentration when compared to the MUHW phenotype.

#### 2.2.5. MHO vs. MUO Phenotype

In middle-aged women with obesity, the comparison of the heathy and unhealthy phenotypes showed differences in the VAT [196 (155–235) vs. 201 (153–227) cm^2^; *p* = 0.001]. There were no differences in the other variables (Table 3).

### 2.3. Associations of Adiponectin, OCN, Irisin, FGF-21 and MCP-1, BMI, VAT, Insulin and HOMA-IR

Osteocalcin showed a negative correlation with BMI (r = −0.107; *p* = 0.047); adiponectin had a negative correlation with BMI (r = −0.217; *p* = 0.001), insulin (r = −0.415; *p* = 0.0001), HOMA-IR (r = −0.429; *p* = 0.0001), and VAT (r = −0.134; *p* = 0.025); irisin had a positive correlation with BMI (r = 0.604; *p* = 0.001), insulin (r = 0.446; *p* = 0.0001), HOMA-IR (r = 0.452; *p* = 0.0001), and VAT (r = 0.645; *p* = 0.0001); FGF-21 had a negative correlation with insulin (r = −0.337; *p* = 0.030) and HOMA-IR (r = −0.341; *p* = 0.03); MCP-1 had a positive correlation with BMI (r = 0.481; *p* = 0.0001), VAT (r = 0.497; *p* = 0.001), insulin (r = 0.298; *p*= 0.001), and HOMA-IR (r = 0.255; *p* = 0.004). The graphical representation of the correlations is shown as Supplementary Material Figures S1–S4.

### 2.4. Adiponectin, OCN, Irisin, FGF-21 and MCP-1 According to Menopausal Status

The effects of menopausal status on the different phenotypes, although they had values above the unit, were not statistically significant (OR of 2.67(_95%_CI 0.94–7.6) in MHNW vs. MUNW; 2.7 (_95%_CI 0.96–7.73) in MHNW vs. MHO; 2.34 (_95%_CI 0.80–6.85) in MHNW vs. MUO, and 0.87 (_95%_CI 0.5–1.51) in MHO vs. MUO.)

### 2.5. Factors Associated with a Metabolic Phenotype

A multiple logistic regression analysis was performed to estimate the factors associated with a metabolic phenotype, which was adjusted according to menopausal status. OCN and adiponectin were significant biomarkers in the comparison of MHNW vs. MUNW and MHNW vs. MHO, irisin for MHNW vs. MUO, and MCP-1 and irisin for MUNW vs. MUO (Table 3). The biomarker that was not significantly associated in any comparison was FGF-21. There was no significant biomarker observed in the comparison of MHO vs. MUO.

## 3. Discussion

In this study, we evaluated the profiling of the adipokine known as adiponectin, the osteokine osteocalcin, the myokine irisin, the hepatokine FGF-21, and the inflammatory cytokine MCP-1, according to the body size phenotype of middle-aged women; likewise, we evaluated the differences between these groups. We corroborated a negative association of osteocalcin with BMI, of adiponectin with BMI, insulin, HOMA-IR and VAT, and of FGF-21 with HOMA-IR, together with a positive association of irisin with BMI, insulin, HOMA-IR and VAT.

Adipose tissue has been recognized as an endocrine and metabolically active organ. Adipose dysfunction, or adiposopathy, is characterized by the deposition of ectopic fat and a shift to visceral adipose tissue distribution, inflammatory and adipokine dysregulation, and insulin resistance. Adiposopathy contributes to cardiovascular and metabolic disease and may explain the heterogeneity of obesity phenotypes, as well as the unhealthy phenotype observed in non-obese individuals [11].

There is an individual variation in the body’s response to excess energy accumulation. There are differences in the gene expression, inflammatory milieu, and the development of traditional risk factors between visceral or subcutaneous fat distribution. The susceptibility to store fat either subcutaneously or viscerally is, in part, determined by genetics [11]. Excess VAT is associated with atherogenic dyslipidemia, hyperinsulinemia, adipocytokine dysfunction, and glucose intolerance. The visceral adiposity index has been found to be the best predictor of an unhealthy metabolic phenotype and is independent of nutritional status (even in normal weight and overweight individuals) and sex [12]. In addition, BMI does not determinate the visceral or subcutaneous fat distribution. An individual with a lower BMI may have a higher VAT when compared to an individual with a higher BMI [11]. These facts may explain the presence of MUNW and MOH phenotypes. MUNW is related with an increased risk of CVD (1.5–3-fold higher than MHNW); this risk is even higher than that observed in MHO [9].

On the other hand, MHO is characterized by a 2.6-fold lower visceral fat deposition, a lower liver fat mass, a higher leg fat content, greater insulin sensitivity, normal inflammation markers, and a preserved adipose tissue function when compared to individuals with MUO [6]; however, MHO is said to be a transient state [6,13] and despite what might be expected, individuals with this phenotype also have a higher risk of developing major vascular events, which shows that obesity remains a major risk factor for cardiovascular disease which is independent of other metabolic factors [13]. VAT (OR 1.99; _95%_CI 1.17–3.39; *p* = 0.01) is associated with the conversion of MHO to an unhealthy phenotype [14]. In our study, we corroborated that the VAT was higher in women with obesity; however, in normal weight women, those with a metabolically unhealthy phenotype had a higher amount of VAT than metabolically healthy women, and VAT showed an association with some cytokines, such as adiponectin, irisin, and MCP-1.

The role of some osteokines and adipokines in obesity and metabolic syndrome has been described; however, the pattern of osteokines, myokines, hepatokines, adipokines, and inflammatory cytokines according to the body size phenotype has been explored little in women.

OCN, a classic marker of bone formation, is involved in the regulation of energy metabolism; its undercarboxylated form (ucOCN) regulates insulin and adiponectin secretion [15].

OCN mRNA and all the genes involved in OCN carboxylation are present in subcutaneous and omental adipose tissue during all the stages of adipogenesis, and both tissues are able to release cOCN and ucOCN. Likewise, a negative correlation has been found between the ucOC/OCN ratio and BMI, which explains why overweight and obese patients have a lower ucOCN and ucOC/OCN ratio [15].

Some meta-analyses have observed that an increased total OCN and ucOCN are correlated with lower BMI and body fat percentage [16,17]. This association is specific to both ethnicity and the presence of obesity [16]. In healthy postmenopausal women, OCN has been considered as a marker of metabolic risk, which has a negative correlation with fasting plasma glucose, and lower concentrations of this osteokine being observed in obese women [18]. In our series, we found that the MUNW and MHO phenotypes had higher OCN concentrations when compared to MHNW phenotype, although specifically in those middle-aged women with an unhealthy phenotype, those with obesity had a lower OCN when compared to normal weight women. OCN had a negative association with BMI, coinciding with that reported in the general population [16,17] and postmenopausal women [18]; additionally, this elevation of OCN was an associated factor for the metabolically unhealthy phenotype in normal weight women.

Adiponectin, a peptide which is predominantly expressed in white adipose tissue, is negatively associated with VAT. Adiponectin increases free fatty acid (FFA) oxidation and glucose uptake, protects hepatocytes from apoptosis, has an anti-inflammatory effect, and reduces FFA influx, de novo lipogenesis, and gluconeogenesis [1,2]. In a cohort of obese and non-obese Mestizo subjects aged 18–70 years old, adiponectin was associated with the MHO phenotype [19].

In our series, adiponectin was lower in the MUNW, MHO and MUO phenotypes when compared to the MHNW phenotype, and in specifically the middle-aged women with an unhealthy phenotype, adiponectin was lower in those with obesity when compared to normal weight women. Adiponectin was negatively associated with BMI, insulin, HOMA-IR, and VAT; additionally, the reduction of adiponectin was an associated factor for a metabolically unhealthy phenotype in normal weight women. To our knowledge, this specific association has not been previously reported in women; however, it is consistent with the conclusion of a meta-analysis that proposed adiponectin as a diagnostic biomarker to identify subjects with metabolic syndrome, especially in high-risk populations with insulin resistance [20].

Irisin, a myokine that is produced by the proteolytical cleavage of fibronectin type III domain-containing 5 (FNDC5), has a critical role in energetic homeostasis. Irisin promotes white adipose tissue browning, increases glucose uptake in muscle, decreases gluconeogenesis, lipogenesis, and lipid accumulation, and regulates β-cell function [2,21]. There is evidence that patients with metabolic syndrome have lower irisin and higher circulating C-reactive protein (CRP) and IL-6; likewise, individuals with central obesity have lower irisin concentrations when compared to individuals without central obesity. In these individuals, serum irisin is negatively associated with systolic BP and fasting plasma glucose and is positively associated with HDL-C concentrations [22]. In our study, MUO women had a higher irisin when compared to MHNW women. This data coincides with a meta-analysis that showed higher circulating irisin in obese individuals when compared to healthy controls [23]. On the other hand, we corroborated that, among women with an unhealthy phenotype, those with obesity had a higher irisin when compared to normal weight women. A positive association was found between irisin and BMI, insulin, HOMA-IR, and VAT, which, to our knowledge, has not yet been reported in specific populations such as, in our case, women.

It has been proposed that the “hyper-irisinemia” that is observed in obesity could be a compensatory mechanism of a resistance to irisin [20]. Specifically, in women, there is a lack of information about irisin concentrations and its characteristics according to body phenotype; however, there is evidence that middle-aged women with osteoporosis have lower concentrations of irisin, which positively correlates with bone mineral density [24].

FGF-21, a member of the FGF-19 subfamily, is involved in the regulation of glucose, lipid, and energy metabolism [25]. FGF-21 is a mediator of the fasting state and contributes to the regulation of lipolysis in white adipose tissue, increases fatty acid oxidation in the liver, and promotes insulin-independent glucose uptake in adipocytes, which improves glucose tolerance and reduces serum triglycerides [26]. FGF-21 has been proposed as a therapeutic target in obesity and insulin resistance [27]. The administration of recombinant FGF-21 in non-human models has been associated with an improved lipoprotein profile, increased glucose tolerance and insulin sensitivity, and reduced hepatic steatosis and obesity [25]. There is evidence that obesity is a state of FGF-21 resistance, both at the signaling and transcriptional level, that is characterized by an increase in FGF-21, but without the biological effects of this hepatokine [26]. In our series, FGF-21 was observed to be lower in both the MHO and MUO phenotypes when compared to the MHNW phenotype. We observed an inverse association between FGF-21, insulin, and HOMA-IR, which could be explained by the effects of this hepatokine on insulin sensitivity.

Lastly, MCP-1, a member of the chemokine (chemotactic cytokine) family, plays a crucial role in a macrophage’s recruitment of adipose tissue. MCP-1 is related to oxidative stress, lipid oxidation, obesity, insulin resistance, and hepatic steatosis [1]. There is evidence that insulin increases the MCP-1 gene and protein expression in insulin-resistant individuals to a greater extent than in insulin-sensitive individuals, and following this stimulation, serum MCP-1 decreases significantly in insulin-sensitive, but not in insulin-resistant, individuals [28]. It has been reported that individuals with obesity have higher concentrations of MCP-1 when compared to non-obese controls, with a positive association of MCP-1 with CRP and IL-6, and a negative association with HDL-C [29]. However, other series have reported no significant differences in MCP-1 between MHNW, MUHNW, MHO, and MUHO individuals [30]. Specifically, in women, it has only been demonstrated that MCP-1 correlates with BMI, waist circumference, and visceral adipose tissue, in postmenopausal women with subclinical atherosclerosis [7]. In our study, we only found a positive association between MCP-1, BMI, insulin, HOMA-IR, and VAT, which adds new information to that published. In addition, among women with an unhealthy phenotype, MCP-1 was higher in those women with obesity when compared to those of normal weight.

One point that stands out in our study is the small number of women in the metabolically healthy groups who were obtained from the sample, both those of normal weight and obese. A meta-analysis reported an overall prevalence of MHO and MUNW phenotypes of 7.27% and 19.98%, respectively, with the highest MHO prevalence found in American populations, and the highest MUNW prevalence found in European populations [31]. In Mediterranean populations, the prevalence of MHO was 2.2% [32].

In the Mexican population, MHO was found in 23% of a cohort of subjects aged 18–70 years and, specifically, in 25% of women aged 18–70 years, with metabolically unhealthy phenotypes predominating, as in our study [19].

There is a lack of information on the frequencies of unhealthy phenotypes, specifically in middle-aged women, both in other populations and in our own; however, according to the most recent National Health Survey, the reported rates of metabolic diseases in middle-aged women are concerning, with the frequency of hypertension, diabetes, hypercholesterolemia, and obese or overweight bodies being 22.9, 22.6, 35.1, and 10.6%, respectively [33]. The present study may indicate that a large number of these women who have metabolic disease may remain undiagnosed until an intentional search for these disorders is conducted, which may lead to proposals for early detection programs.

The strengths of our study include the fact that this study is the first to recognize differences in the pattern of some osteokines, myokines, hepatokines, adipokines, and inflammatory cytokines between various body size phenotypes in middle-aged women, as well as, to our knowledge, being the first study to demonstrate associations of some of these cytokines with metabolic parameters such as BMI, VAT, or HOMA-IR in this specific population. Also, further clinical studies are needed to clarify the role of these cytokines, because, in many of these subjects, the local tissue concentrations measured may be divergent from the serum level, and the substantial differences between auto- and endocrine effects of these molecules need to be considered.

Limitations of this study include its cross-sectional nature and the possibility of random error, as it is a non-probability study. For future research, we propose to conduct case–control studies, and to intentionally look for differences according to body phenotypes, both in these five cytokines analyzed here, and in other cytokines belonging to each of the groups (e.g., leptin, interleukins, resistin, etc.).

## 4. Materials and Methods

### 4.1. Study Design and Patients

A cross-sectional, non-probabilistic study was performed in 265 middle-aged women aged from 40 to 65 years. The inclusion criteria included Mexican–Mestizo women aged from 40 to 65 years with either obesity or a normal-weight, who were enrolled in the outpatient clinic of the Endocrine Research Unit of the Hospital de Especialidades, of the Centro Médico Nacional, Instituto Mexicano del Seguro Social (IMSS). The exclusion criteria included women with an established diagnosis of diabetes, as well as those with renal or liver failure, were overweight, or who had chronic infections, endocrine or blood disorders, neoplasm, or a history of cardiovascular disease.

### 4.2. Clinical Evaluation

#### 4.2.1. Anthropometry

The participants underwent a clinical examination. The anthropometric indicators of each patient were recorded: body weight, height, and blood pressure were measured. Body mass index (BMI) was calculated by dividing the participants’ weight by the square of their height (kg/m^2^). The participants were classified, according to BMI, into a normal weight or obesity. Their waist circumference was measured with the participant standing, at the midpoint of the distance between the iliac crest and the inferior border of the last rib, at the end of expiration. Body composition was assessed by electrical bioimpedance (Body Composition 353 IOI Analyzer, Jawon Medical Co., Ltd., Gyeonsgsangbuk-do, South Korea) after 12 h of fasting, with adequate hydration. The bioelectric impedance was measured with the patient upright, wearing light clothing, and without shoes. The analyzer measured the participants’ weight to an accuracy of within 0.1 kg, as well as their body impedance (in ohms), with calculation of the visceral adipose tissue (VAT).

#### 4.2.2. Clinical Characteristics and Definitions

A participant was considered of normal weight if their BMI ranged from 18.4 to 25. Obesity was defined as a BMI above 30. To define metabolic health, the Karelis criteria were used [34]: TC ≤ 200 mg/dL, triglycerides ≤ 150 mg/dL, HDL-C ≥ 50 mg/dL, and no treatment, LDL-C ≤ 100 mg/dL and no treatment, and HOMA-IR ≤ 2.8. A metabolically healthy phenotype was defined as the presence of ≥4 Karelis criteria. A diagnosis of menopause was confirmed by a serum estradiol concentration of ≤25 pg/mL as well as amenorrhea for at least one year. None of the patients were receiving hormonal replacement therapy.

### 4.3. Phenotypes

Four phenotypes were assessed according to metabolic health and the presence or absence of obesity: MHNW, MUNW, MHO, and MUO. The comparison between the MHNW vs. MUNW phenotypes, the MHNW vs. MHO phenotypes, the MHNW vs. MUO phenotypes, and the MHO vs. MUO phenotypes was performed. MHNW was considered as the reference group.

### 4.4. Biochemical Evaluation

A biochemical analysis was performed using blood samples collected from the antecubital vein, following the use of standard phlebotomy techniques. All the blood draws were performed in the morning (i.e., 08:00) following an overnight fast (≥12 h). Samples were collected into two tubes without an anticoagulant, centrifuged at 640 g for 15 min, and aliquots of serum were then isolated and prepared for testing. Serum concentrations of glucose, TC, and HDL-C, as well as triglycerides concentrations, were quantified using semiautomatic photometry (semi-automatic chemical analyzer Ekem KontroLab, San Antonio, TX, USA), and the serum LDL-C concentrations were calculated using Friedewald’s formula. Insulin concentrations were determined using a solid-phase radioimmunoassay (Millipore, Billerica, MS, USA). The intra- and inter-assay coefficients of variation (CV) were 3 and 9%, respectively. Insulin resistance was quantified using the homeostasis model assessment (HOMA-IR) (HOMA-IR = insulin (mIU/mL) × fasting glucose (mmol/L)/22.5) [35]. Serum concentrations of osteocalcin were measured using a chemiluminescent immunoassay (Diagnostic Products Corporation, Los Angeles, CA, USA). Enzyme-linked immunosorbent assays (ELISA) were used for the quantitative determination of adiponectin (Linco Research Lnc., Street Charles, Mo, USA), irisin (Cloud-clone corps. Katy, TX, USA, Catalog No. SEN576Hu), MCP-1 (Catalog No. MBS355283, MyBioSource Inc, San Diego, CA, USA), and FGF-21 (Catalog No. EK-073-33, Phoenix Pharmaceuticals Inc, Burlingame, CA, USA). The intra- and inter-assay CV of adiponectin, irisin, MCP-1 and FGF-21 were 5 and 8, 4 and 7, 5 and 5, and 4 and 9.0%, respectively.

### 4.5. Statistical Analysis

Parametric variables were expressed as the mean ± standard deviation and non-parametric were expressed as the median and interquartile range (IQR). Differences between groups were evaluated by either the Student *t*-test or the Mann–Whitney U test, or the Wilcoxon signed-rank test, as appropriate. The correlation among variables was identified by the Pearson’s or Spearman’s tests. A multiple logistic regression analysis was performed to estimate the factors that were associated with a metabolic phenotype, which was adjusted by menopausal status, VAT, OCN, adiponectin, irisin, FGF-21, and MCP-1. All analyses were performed with the statistical package SPSS v.21. A statistically significant *p* value was considered as *p* < 0.05.

The required sample size (264) was met (alpha value of 0.05, power of 95%, maximum ratio of 0.50 and precision of 3%).

### 4.6. Ethics

The study was conducted according to the guidelines of the Declaration of Helsinki and approved by the Ethics Committee of Instituto Mexicano del Seguro Social (IMSS) (protocol no. R-2016-3601-191, approved in 2016 and re-approved on 27 December 2018). Subjects who agreed to participate signed an informed consent form.

## 5. Conclusions

The concentrations of adiponectin, osteocalcin, irisin, FGF-21, and MCP-1 may vary according to the body size phenotype in middle-aged women. Our study shows that these cytokines are associated with some metabolic parameters such as BMI, insulin, HOMA-IR and VAT in Mexican–Mestizo middle-aged women. The assessment of the pattern of some osteokines, myokines, hepatokines, adipokines, and inflammatory cytokines revealed that these could be biomarkers of an unhealthy metabolic phenotype, one that is independent of the classical measurement of glucose, lipid profile and the determination of HOMA-IR in middle-aged women. However, further studies in different populations are needed to determinate its usefulness in clinical practice.

Early detection of pathogenesis through biomarkers holds the key to preventing cardiovascular complications, as well as furthering knowledge of the risk of metabolic pathologies in various body size phenotypes among middle-aged women, as suggested by the results of this study through cytokine assessment.

## Figures and Tables

**Table 1:** Baseline characteristics of the middle-aged women included in the study.

Characteristics	Results
Age; (years)	52.2 ± 5.8
Postmenopausal; % (*n*=)	67.9 (180)
BMI; (kg/m^2^)	28.8 ± 4.9
Phenotype; % (*n*=)	MHNW: 6 (16) MUNW: 57 (151) MHO: 1.9 (5) MUO: 35.1 (93)
VAT; (cm^2^)	127 (98–167)
Osteocalcin; (ng/mL)	5.2 (3.5–7.7)
Adiponectin; (µg/mL)	11.7 (7.8–16.0)
Irisin; (ng/mL)	495.4 (267.9–713.2)
FGF-21; (pg/mL)	205.0 (73.8–353.0)
MCP-1; (pg/mL)	21.7 (8.1–37.4)
Glucose; (mg/dL)	85 (77–95)
Insulin; (mIU/L)	17 (13–24)
HOMA-IR	3.5 (2.6–5.3)
Total cholesterol; (mg/dL)	225.5 (200.5–267.75)
LDL-C; (mg/dL)	141.8 (118.4–170.6)
HDL-C; (mg/dL)	53.5 (45–63)
Triglycerides; (mg/dL)	142 (111–198)

Parametric variables are represented as mean ± standard deviation. Non-parametric variables are represented as median (interquartile range). BMI: body mass index. VAT: visceral abdominal tissue. HOMA-IR: homeostasis model assessment of insulin resistance. LDL-C: low-density cholesterol. HDL-C: high-density cholesterol. FGF-21: fibroblast growth factor 21. MCP-1: monocyte chemoattractant protein-1.

**Table 2:** Clinical and biochemical characteristics according to the body size phenotype in middle-aged women.

	MHNW (*n* = 16)	MUNW (*n* = 151)	MHO (*n* = 5)	MUO (*n* = 93)	MHNW vs. MUNW *p* Value	MHNW vs. MHO *p* Value	MHNW vs. MUO *p* Value	MUNW vs. MUO *p* Value	MHO vs. MUO *p* Value
Age (years)	51.4 ± 7.1	52.7 ± 5.7	53 ± 5.3	51.6 ± 5.6	0.380	0.375	0.875	0.14	0.26
VAT; (cm^2^)	91.5 (68–123)	105 (87–125)	195.6 (155–215)	201 (153–227)	0.112	0.080	0.0001 *	0.0001 *	0.001 *
Osteocalcin; (ng/mL)	3.4 (2.5–5.5)	5.8 (3.7–8.4)	5.2 (3.8–6.8)	4.7 (3.4–7.0)	0.011 *	0.011 *	0.157	0.001 *	0.761
Adiponectin; (µg/mL)	16.1 (11.2–21.7)	12.5 (7.9–16.0)	14.6 (9.8–29.1)	10.1 (7.6–13.3)	0.002 *	0.002 *	0.0001 *	0.03 *	0.155
Irisin; (ng/mL)	464 (186–559)	348.8 (201.4–660)	649 (270–868)	676 (503–787)	0.909	0.413	0.0001 *	0.0001 *	0.942
FGF-21; (pg/mL)	370 (315–851)	201 (70–335)	195 (194–197)	162 (21.8–229)	0.067	0.024 *	0.004 *	0.30	0.279
MCP-1; (pg/mL)	15.9 (4.3–43.5)	15.8 (7.7–34.2)	25.1 (18.5–46.5)	38.6 (32.7–52.8)	0.620	0.756	0.291	0.0001 *	0.627
Glucose; (mg/dL)	74 (64–85)	83 (77–91)	77 (74–91)	89 (79.5–98.5)	0.003 *	0.004 *	0.0001 *	0.007 *	0.121
Insulin; (mIU/L)	12.1 (10–13.2)	15.8 (12.6–20.5)	22.5 (12.4–34.4)	22.5 (17.9–30.2)	0.0001 *	0.0001 *	0.0001 *	0.0001 *	0.62
HOMA-IR	2.14 (1.5–2.6)	3.2 (2.4–4.7)	4.2 (2.2–7.9)	4.9 (3.7–7.4)	0.0001 *	0.0001 *	0.0001 *	0.0001 *	0.342
Total cholesterol; (mg/dL)	186 (175–190)	230 (211–275)	165 (124.5–178.5)	229 (203–262)	0.0001 *	0.0001 *	0.0001 *	0.237	0.001 *
LDL-C; (mg/dL)	95 (87–107)	145 (124–178)	62.6 (54.2–92.6)	147 (124–179)	0.0001 *	0.0001 *	0.0001 *	0.65	0.001 *
HDL-C; (mg/dL)	67 (54–72)	55 (45–63)	63.0 (47.5–71.0)	49 (41–59)	0.002 *	0.002 *	0.0001 *	0.01 *	0.09 *
Triglycerides; (mg/dL)	109 (80–125)	145 (109–198)	121 (88.5–138)	154 (121–209)	0.003 *	0.003 *	0.0001 *	0.20	0.056 *

Parametric variables are represented as mean ± standard deviation. Non-parametric variables are represented as median (interquartile range). BMI: body mass index. VAT: visceral abdominal tissue. HOMA-IR: homeostasis model assessment of insulin resistance. LDL-C: low density cholesterol. HDL-C: high density cholesterol. FGF-21: fibroblast growth factor 21. MCP-1: monocyte chemoattractant protein-1. * Statistically significant *p* < 0.05.

**Table 3:** Multiple logistic regression model adjusted by menopausal status according to the different phenotypes.

	MHNW vs. MUNW OR (_95%_CI)	MHNW vs. MHO OR (_95%_CI)	MHNW vs. MUO OR (_95%_CI)	MUNW vs. MUO OR (_95%_CI)
Osteocalcin; (ng/mL)	1.4 (1.06–1.81)	1.4 (1.07–1.84)	NS	NS
Adiponectin; (µg/mL)	0.9 (0.84–0.96)	0.9 (0.84–0.96)	NS	NS
Irisin; (ng/mL)	NS	NS	1.01 (1.001–1.01)	1.007 (1.003–1.011)
MCP-1; (pg/mL)	NS	NS	NS	1.044 (1.008–1.083)

Model adjusted by menopausal status, VAT, OCN, adiponectin, irisin and MCP-1. VAT: visceral abdominal tissue. FGF-21: fibroblast growth factor 21. MCP-1: monocyte chemoattractant protein-1. NS. Non-significant. Comparisons between groups with significant values are shown.

## Data Availability

The data presented in this study are available on request from the corresponding author.

## Supplementary Materials

The following are available online at https://www.mdpi.com/article/10.3390/metabo11110771/s1, Figure S1: Correlation between adiponectin, irisin, MCP-1 and visceral adipose tissue, Figure S2: Correlation between levels of adiponectin, irisin and MCP-1, FGF-21 and HOMA-IR, Figure S3: Correlation between adiponectin, irisin, MCP-1, FGF-21 and insulin levels, Figure S4: Correlation between osteocalcin, adiponectin, irisin, MCP-1 levels and BMI.

## Abbreviations

BMI	body mass index
CRP	C-reactive protein
CV	coefficients of variation
CVD	cardiovascular disease
FFA	free fatty acid
FGF-21	fibroblast growth factor-21
FNDC5	fibronectin type III domain-containing 5
HDL-C	high density cholesterol
HOMA-IR	homeostasis model assessment of insulin resistance
IL-1β	interleukin-1β
IL-6	interleukin-6
IQR	interquartile range
LDL-C	low density cholesterol
MCP-1	monocyte chemoattractant protein-1
MHNW	metabolically healthy normal weight
MHO	metabolically healthy obesity
MUNW	metabolically unhealthy normal weight
MUO	metabolically unhealthy obesity
OCN	osteocalcin
TC	total cholesterol
TNF-α	tumor necrosis alpha
ucOCN	undercarboxylated osteocalcin
VAT	visceral adipose tissue
