# Supplementary material for: Pattern of Adiponectin, Osteocalcin, Irisin, FGF-21, and MCP-1 According to the Body Size Phenotype: Could They Be Markers of Metabolic Health in Mexican-Mestizo Middle-Aged Women?
Source: Metabolites. 2021 Nov 11;11(11):771. doi: 10.3390/metabo11110771 (PMC8619823; doi:10.3390/metabo11110771)
Supplement: Supplementary file 1 [file metabolites-11-00771-s001.zip › Basurto et al. Supplementary Material 11.11.21.pdf]

Fig. S1

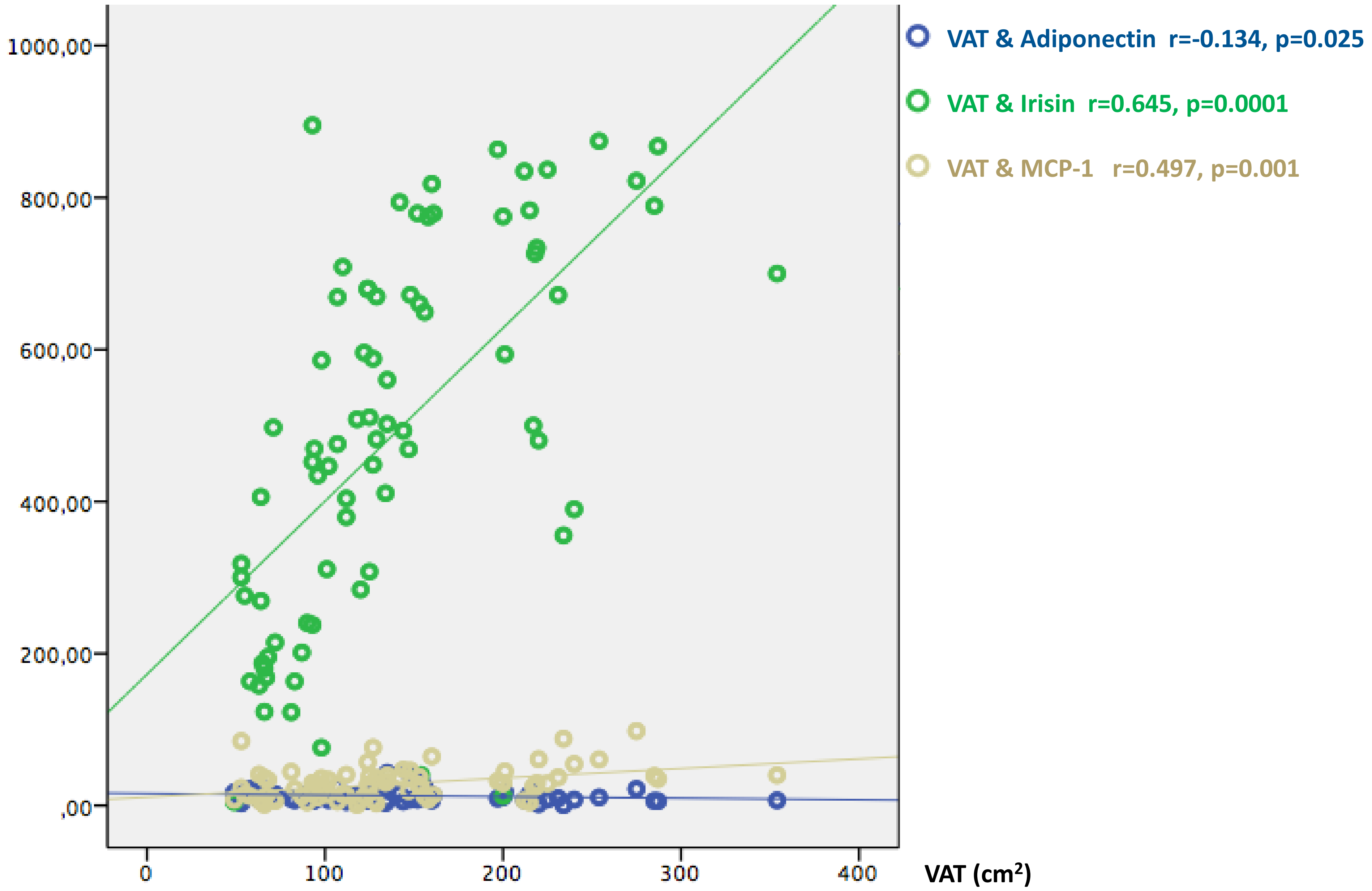

Figure S1. Correlation between adiponectin, irisin, MCP-1 levels and VAT.

MCP-1: monocyte chemoattractant protein-1 , VAT: visceral adipose tissue.

Fig. S2

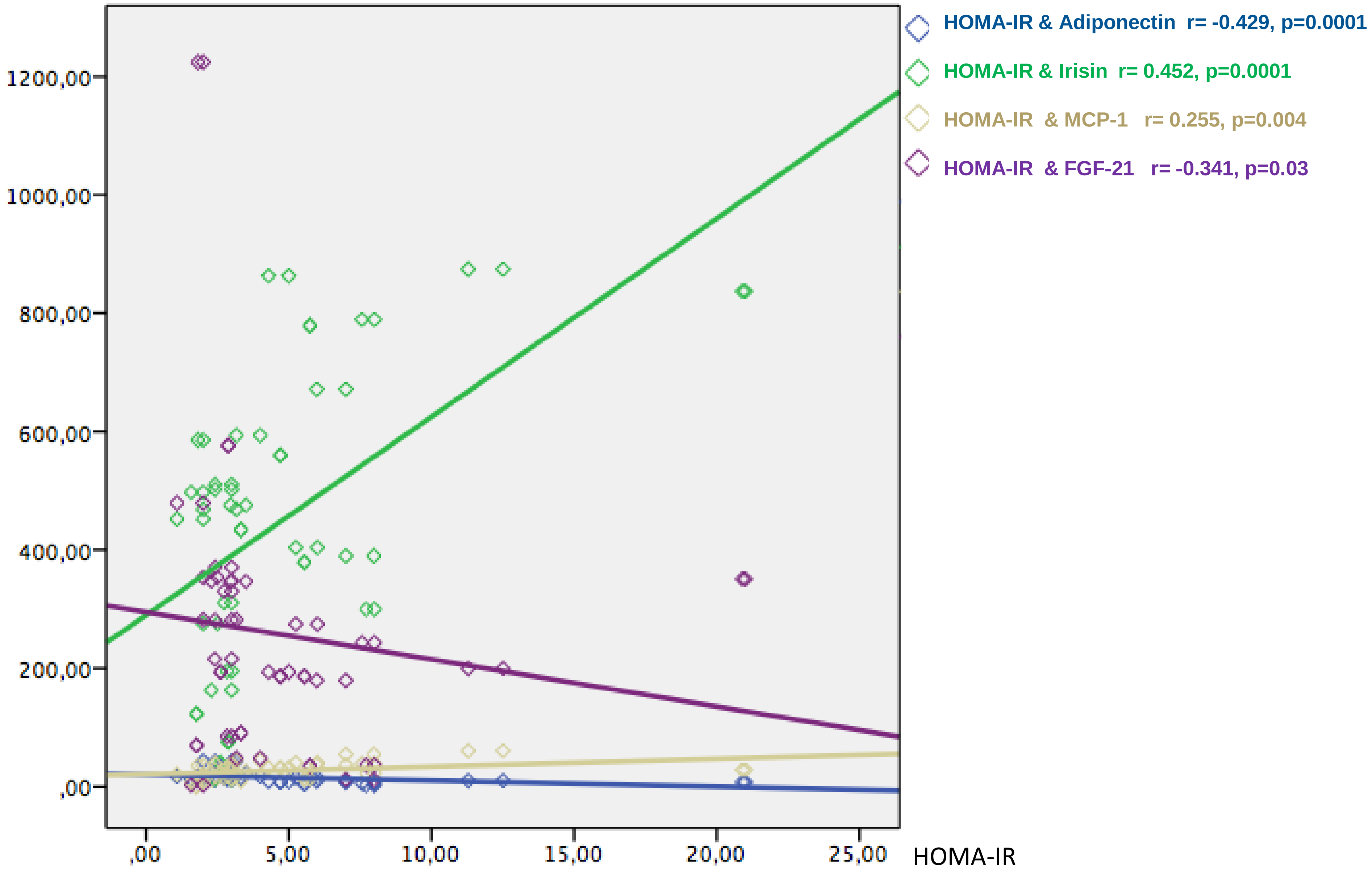

Figure S2. Correlation between adiponectin, irisin, MCP-1, FGF-21 levels and HOMAR-IR.

MCP-1: monocyte chemoattractant protein-1, , FGF-21: fibroblast growth factor-21.

Fig. S3

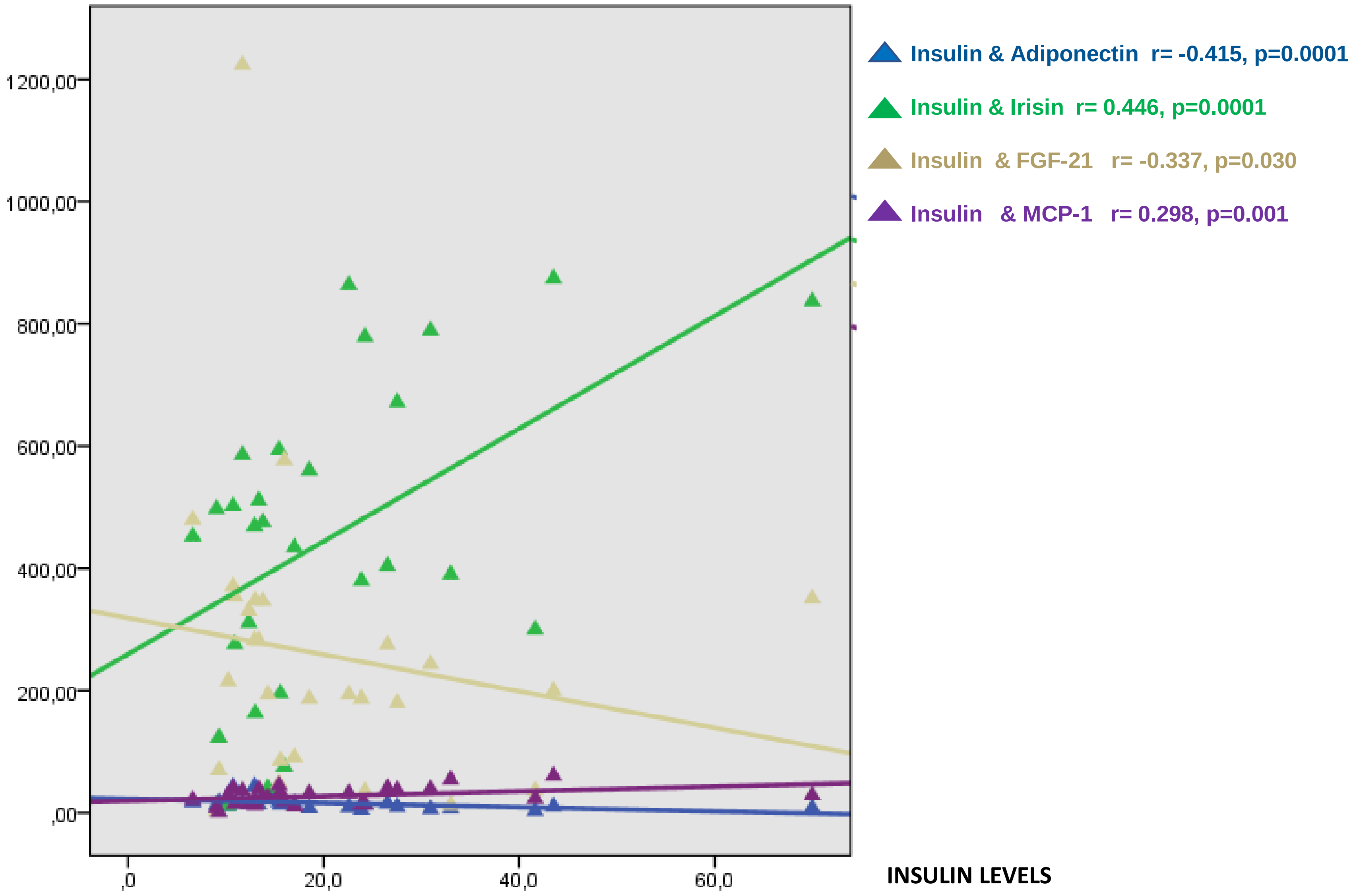

Figure S3. Correlation between adiponectin, irisin, MCP-1, FGF-21 and insulin levels.

MCP-1: monocyte chemoattractant protein-1, FGF-21: fibroblast growth factor-21.

Fig. S4

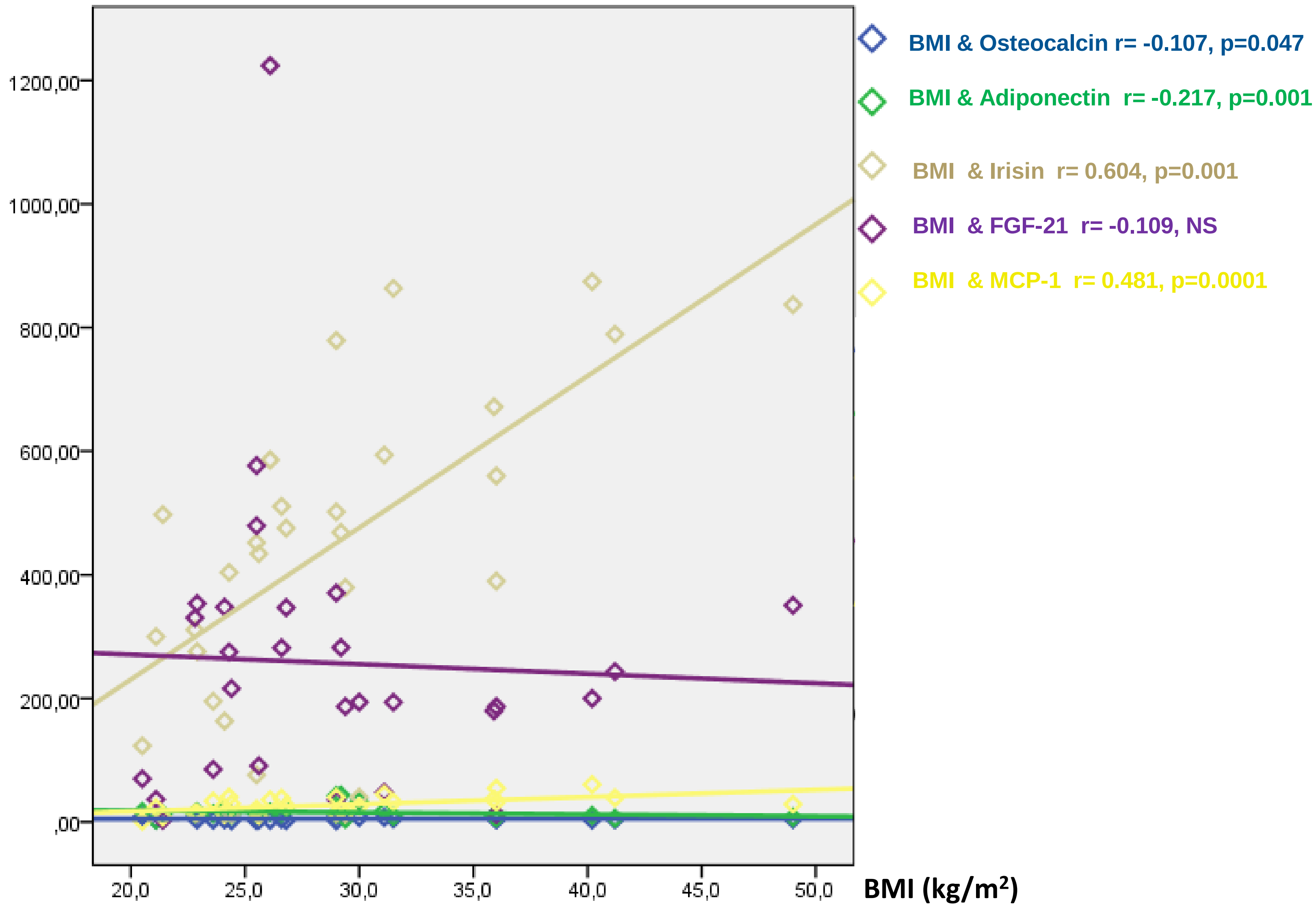

Figure S4. Correlation between osteocalcin, adiponectin, irisin, FGF-21, MCP-1 levels and BMI.

MCP-1: monocyte chemoattractant protein-1, FGF-21: fibroblast growth factor-21, BMI: body mass index.
